# Impact of Yuehua Decoction on quality of life and safety in multidrug-resistant tuberculosis: a prospective cohort study using multivariable regression analysis

**DOI:** 10.3389/fmed.2026.1801335

**Published:** 2026-04-16

**Authors:** Qingfeng Sun, Qiwen Huang, Xin Mai, Lan Mo, Yunjie Qin, Liyuan Li, Yanzhen Zeng, Qianyu Liu, Yan Liao, Aimei Liu

**Affiliations:** 1 Department of Tuberculosis, Guangxi Zhuang Autonomous Region Chest Hospital, Liuzhou, Guangxi, China; 2 Department of Traditional Chinese Medicine, Guangxi Zhuang Autonomous Region Chest Hospital, Liuzhou, Guangxi, China; 3 Care Clinic, Guangxi Zhuang Autonomous Region Chest Hospital, Liuzhou, Guangxi, China

**Keywords:** cohort study, multidrug-resistant tuberculosis, quality of life, traditional Chinese medicine, Yuehua Decoction

## Abstract

**Background:**

Multidrug-resistant tuberculosis (MDR-TB) remains a major global health challenge, and prolonged chemotherapy often leads to substantial symptom burden and impaired health-related quality of life (HRQoL). Host-directed adjunctive therapies, including Traditional Chinese Medicine, have received growing interest; however, prospective clinical evidence on Yuehua Decoction remains limited. This study evaluated whether adjunctive Yuehua Decoction improves HRQoL and safety among patients receiving standardized MDR-TB treatment.

**Methods:**

We conducted a prospective, non-randomized cohort study enrolling adults with microbiologically confirmed MDR-TB. Patients received either standard MDR-TB chemotherapy alone (control group) or in combination with Yuehua Decoction (Yuehua group) based on treatment preference. The primary outcome was HRQoL at 6 months, assessed using the St. George’s Respiratory Questionnaire (SGRQ). Between-group differences were analyzed using multivariable linear regression adjusting for baseline SGRQ and other clinically relevant covariates. Secondary outcomes included sputum culture conversion and treatment-related adverse events.

**Results:**

Seventy-one patients were included (Yuehua group, *n* = 32; control group, *n* = 39). Although demographic characteristics were similar, baseline SGRQ scores were significantly higher in the Yuehua group (*P* = 0.007). After adjustment for this imbalance, adjunctive Yuehua Decoction was associated with greater improvement in SGRQ total score at 6 months (adjusted β = –3.00; 95% CI: –3.87 to –2.12; *P* < 0.001). The between-group difference was slightly below the conventional minimal clinically important difference (4 points), but the direction and precision of the estimate suggest potential clinical relevance. Rates of sputum culture conversion and overall treatment success were comparable between groups. A significantly lower incidence of skin reactions was observed in the Yuehua group (15.6% vs. 35.9%, *P* = 0.038), with no increase in serious adverse events.

**Conclusion:**

In this preliminary prospective cohort, adjunctive Yuehua Decoction was associated with improved HRQoL without increasing treatment-related toxicity among patients undergoing MDR-TB chemotherapy. These findings support Yuehua Decoction as a potential host-directed adjunct, but confirmation in larger randomized controlled trials is needed.

## Background

Multidrug-resistant tuberculosis (MDR-TB), defined as tuberculosis resistant to at least isoniazid and rifampicin, remains a major threat to global tuberculosis control (1). According to the Global Tuberculosis Report 2025, an estimated 390,000 individuals developed multidrug- or rifampicin-resistant tuberculosis (MDR/RR-TB) worldwide in 2024, yet only 42% were enrolled in treatment, and treatment success rates, though improving to 71%, remain below targets despite expanded diagnostic coverage and the rollout of shorter all-oral regimens (2). Prolonged multi-drug chemotherapy, which typically extends for 18 months or longer, continues to impose substantial physical, psychological, and socioeconomic burdens on affected patients (3, 4). Beyond achieving microbiological cure, improving patient-centered outcomes has become an increasingly important priority in MDR-TB management (5).

Health-related quality of life (HRQoL) in MDR-TB patients is frequently impaired due to chronic symptoms, delayed sputum conversion, medication toxicities, social stigma, malnutrition, and emotional distress (6, 7). Studies using validated instruments such as the St. George’s Respiratory Questionnaire (SGRQ) have demonstrated persistently elevated scores even after bacteriologic improvement, indicating residual functional limitation and poor perceived health status (8, 9). Standard chemotherapy primarily targets the pathogen and may not adequately address host inflammation, oxidative stress, immune dysregulation, or long-term symptom burden (10, 11). Therefore, interest has grown in host-directed adjunctive therapies aimed at improving tolerance, resilience, and recovery (12).

Traditional Chinese Medicine (TCM), as a long-standing host-modulating therapeutic system, has been increasingly explored within the host-directed therapy framework (13, 14). In clinical practice, many MDR-TB patients present with patterns characterized by Yin deficiency, impaired Lung–Kidney function, and Qi consumption secondary to chronic infection and prolonged pharmacotherapy (15). Yuehua Decoction, a classical TCM formulation used in tuberculosis care, integrates herbs such as Glehnia littoralis and Ophiopogon japonicus, which have been shown in pharmacological studies to exert immunomodulatory, anti-inflammatory, and antioxidative effects (16–18). Small observational studies have reported symptomatic improvement and reduced drug-related discomfort among TB patients receiving Yuehua-based treatments (19, 20). However, these studies were often limited by heterogeneous populations, nonstandardized HRQoL assessment, and lack of prospective design.

Despite its widespread empirical use, rigorous clinical evidence evaluating Yuehua Decoction as an adjunct to contemporary MDR-TB regimens remains scarce. In our setting, contemporary MDR-TB therapy consists of individualized all-oral regimens built around bedaquiline, typically combined with a fluoroquinolone (levofloxacin or moxifloxacin), linezolid, cycloserine, and/or clofazimine, in accordance with WHO consolidated guidelines (21). To our knowledge, no prospective study has evaluated a defined Yuehua Decoction formula using validated SGRQ-based HRQoL endpoints in this treatment context. Previous investigations were generally limited by retrospective designs, non-standardized outcome assessment, and heterogeneous populations (22).

Importantly, the clinical relevance of Yuehua Decoction may extend beyond general symptomatic relief. Its Yin-nourishing and anti-inflammatory constituents—particularly Glehnia littoralis, Ophiopogon japonicus, and Stemona sessilifolia—have demonstrated the capacity to suppress pro-inflammatory cytokines and modulate immune hypersensitivity responses in preclinical studies (17, 18, 23, 24). These properties suggest potential benefits in two clinically important domains: first, attenuation of respiratory symptoms such as cough, dyspnea, and fatigue, which are the principal drivers of impaired SGRQ scores in tuberculosis patients (8, 9); and second, reduction of drug-related dermatologic adverse events, which are a recognized barrier to treatment adherence in bedaquiline- and clofazimine-containing regimens (25, 26). However, these mechanistic hypotheses have not been tested in a prospective clinical study of MDR-TB patients.

To address this knowledge gap, we conducted a prospective, non-randomized cohort study to assess the impact of adjunctive Yuehua Decoction on HRQoL among patients undergoing standardized MDR-TB treatment. The primary objective was to estimate the adjusted between-group difference in SGRQ total score at 6 months, controlling for baseline SGRQ score and clinically relevant confounders. Secondary objectives included comparison of sputum culture conversion, treatment success, and treatment-related adverse events. Our findings aim to provide preliminary clinical evidence supporting the potential role of Yuehua Decoction within host-directed care strategies for MDR-TB.

## Materials and methods

### Study design and setting

This study was designed as a prospective observational cohort study conducted in Guangxi, China. The study was carried out in accordance with the principles of the Declaration of Helsinki. The protocol was approved by the Ethics Committee [Approval No.: (No. 2022-012)] and was prospectively registered with the International Traditional Medicine Clinical Trial Registry (ITMCTR; Registration No. ITMCTR2025002072).

### Participants

Patients were recruited between July 1, 2022, and June 30, 2023. Eligible participants were adults (aged ≥ 18 years) with a confirmed diagnosis of MDR-TB according to national and WHO guidelines (15). Inclusion criteria further required that patients be suitable for Yuehua Decoction treatment based on TCM syndrome differentiation, specifically Yin deficiency with Lung-Heat, diagnosed by two experienced TCM practitioners (27). Patients were excluded if they had: (1) extrapulmonary tuberculosis (excluding tuberculous pleurisy); (2) severe or uncontrolled comorbidities (e.g., severe cardiac disease, malignancy, HIV coinfection, or uncontrolled diabetes mellitus); (3) severe psychiatric disorders affecting treatment adherence; (4) pregnancy or lactation; or (5) a life expectancy of less than 6 months.

### Interventions

Treatment group assignment was non-randomized. All eligible patients meeting both the MDR-TB diagnostic criteria and the TCM syndrome differentiation criteria (Yin deficiency with Lung-Heat) were informed of both treatment options during the enrollment visit. Group assignment was then determined by patient preference, with clinical input from the treating physician; no patients were assigned based on disease severity or prognosis. This non-randomized design introduces the possibility of confounding by indication, which is addressed in the Statistical Analysis and Limitations sections.

Patients in the control group received standardized MDR-TB chemotherapy in accordance with the WHO consolidated guidelines (21). The background regimen generally included a fluoroquinolone (such as levofloxacin or moxifloxacin), bedaquiline, linezolid, cycloserine, and/or clofazimine, with final drug selection and dosing individualized according to drug susceptibility testing and clinical evaluation. Treatment duration followed guideline recommendations.

Patients in the Yuehua Decoction group received the same standardized chemotherapy regimen, supplemented with Yuehua Decoction provided and quality-controlled by the Department of Traditional Chinese Medicine. The formula consisted of Glehnia littoralis (Bei Shashen, 12 g), Ophiopogon japonicus (Maidong, 12 g), Asparagus cochinchinensis (Tiandong, 12 g), Rehmannia glutinosa (Sheng Dihuang, 9 g), Rehmannia glutinosa (prepared, Shu Dihuang, 12 g), Equus asinus gelatin (Ejiao, 6 g), Dioscorea opposita (Shanyao, 12 g), Poria cocos (Fuling, 9 g), Morus alba (Sangye, 12 g), Chrysanthemum morifolium (Juhua, 9 g), Stemona sessilifolia (Baibu, 15 g), Panax notoginseng (Sanqi, 9 g), and Fritillaria cirrhosa (Chuanbei, 3 g). All herbs were sourced from GMP-certified commercial suppliers and authenticated by a licensed TCM pharmacist. The decoction was prepared according to a written standard operating procedure (SOP) by hospital pharmacy staff, with batch records maintained; however, formal batch-to-batch chemical fingerprinting (e.g., HPLC) was not performed, which is acknowledged as a limitation. The decoction was administered orally twice daily. Adjunctive therapy was initiated concurrently with anti-tuberculosis chemotherapy during the intensive treatment phase, and the actual duration of intake varied depending on patient tolerance and symptom improvement; however, all patients were observed for a total of 6 months. No additional herbal co-interventions were permitted during the study period.

Anti-TB drug adherence was monitored via directly observed therapy (DOT) during hospitalization and monthly pharmacy dispensing records during outpatient care. Yuehua Decoction adherence was assessed through pharmacy dispensing logs and patient self-report at monthly visits. Patients were considered adherent if they consumed ≥ 80% of prescribed doses.

### Data collection and outcome

Demographic, clinical, radiological, and laboratory information was collected at baseline. Participants were followed for 6 months post-treatment initiation, with clinical status and medication use recorded during monthly visits (scheduled every 30±7 days). The 6-month primary endpoint assessment was specifically conducted at 24±2 weeks.

The primary outcome was health-related quality of life, evaluated using the Chinese-validated version of the St. George’s Respiratory Questionnaire (SGRQ) (28). This 50-item, self-administered tool assesses three distinct domains: Symptoms (frequency and severity), Activity (physical limitations due to breathlessness), and Impacts (social and psychological effects). SGRQ total scores range from 0 to 100, with higher scores reflecting poorer health. A reduction of at least 4 points was defined as the minimal clinically important difference (MCID) (29). We acknowledge that while this MCID is established in COPD populations, its specific applicability to MDR-TB has not been formally validated.

Secondary outcomes included: (1) sputum culture conversion, defined as two consecutive negative cultures obtained at least 30 days apart; and (2) treatment success at 6 months, defined as cure or treatment completion according to WHO criteria (21).

Safety outcomes were evaluated throughout the 6-month observation period. Adverse events were defined as any new or worsening symptom, sign, or laboratory abnormality occurring after treatment initiation. Ascertainment was active: at each monthly visit, patients were systematically asked about predefined events of interest, including hepatotoxicity, skin reactions, gastrointestinal symptoms, arthralgia, and peripheral neuropathy, and underwent routine laboratory monitoring (liver function tests, complete blood count, and electrocardiography including QTc interval). All adverse events were graded for severity using the Common Terminology Criteria for Adverse Events (CTCAE), version 5.0 (30). Causality attribution followed the WHO-UMC system (31). Serious adverse events and events requiring treatment interruption, hospitalization, or regimen modification were independently reviewed by the study clinical team. QTc intervals and liver function were monitored as proxies for potential herb–drug interactions with bedaquiline and linezolid, though formal pharmacokinetic interaction studies were not performed.

### Statistical analysis

Statistical analyses were conducted using Python (version 3.10) with the pandas (v2.0), scipy (v1.11), and statsmodels (v0.14) libraries. Continuous variables were summarized as mean ± standard deviation (SD) or median with interquartile range (IQR), depending on distribution assessed by the Shapiro–Wilk test, and compared using Student’s *t*-test or the Mann–Whitney U test. Categorical variables were presented as frequencies and percentages and compared using the chi-square test or Fisher’s exact test, as appropriate.

To account for baseline imbalances inherent to the non-randomized design and to address potential confounding by indication, multivariable regression models were constructed. Covariates were selected a priori based on clinical prognostic importance and observed baseline imbalance, prior to outcome analysis. For the primary outcome—SGRQ total score at 6 months—multivariable linear regression was performed adjusting for treatment group, baseline SGRQ score, age, and diabetes mellitus. Covariates were selected a priori based on clinical prognostic importance and observed baseline imbalance. Secondary binary outcomes, including sputum culture conversion and treatment success, were analyzed using multivariable logistic regression adjusting for age and diabetes mellitus. Effect estimates were reported as adjusted regression coefficients (β) for linear models and adjusted odds ratios (aORs) for logistic models, each with corresponding 95% confidence intervals (CIs).

Regression assumptions were evaluated through standardized diagnostic procedures. For the linear model, normality and homoscedasticity of residuals were assessed by visual inspection of residual plots. For logistic models, the Hosmer–Lemeshow test was used to assess goodness-of-fit. Multicollinearity was examined using variance inflation factors (VIFs; all <2.0). Full regression output for the primary linear model, including coefficients, standard errors, *P*-values, and VIFs, is provided in Supplementary Table 1.

Missing data were minimal (6.6%; 5 of 76 enrolled patients lacked complete follow-up). A missingness table by group and variable is provided in Supplementary Table 2. The primary analysis used complete-case analysis. To assess robustness, two pre-planned sensitivity analyses were conducted: (1) multiple imputation by chained equations (MICE; *m* = 20 imputations, predictive mean matching) under a missing-at-random assumption; and (2) a worst-case analysis in which missing Yuehua group patients were assigned the highest (worst) observed SGRQ score and missing control patients the lowest (best) observed score. Sensitivity analysis results are reported in Supplementary Table 3.

## Results

### Participant flow and baseline characteristics

**FIGURE 1:**
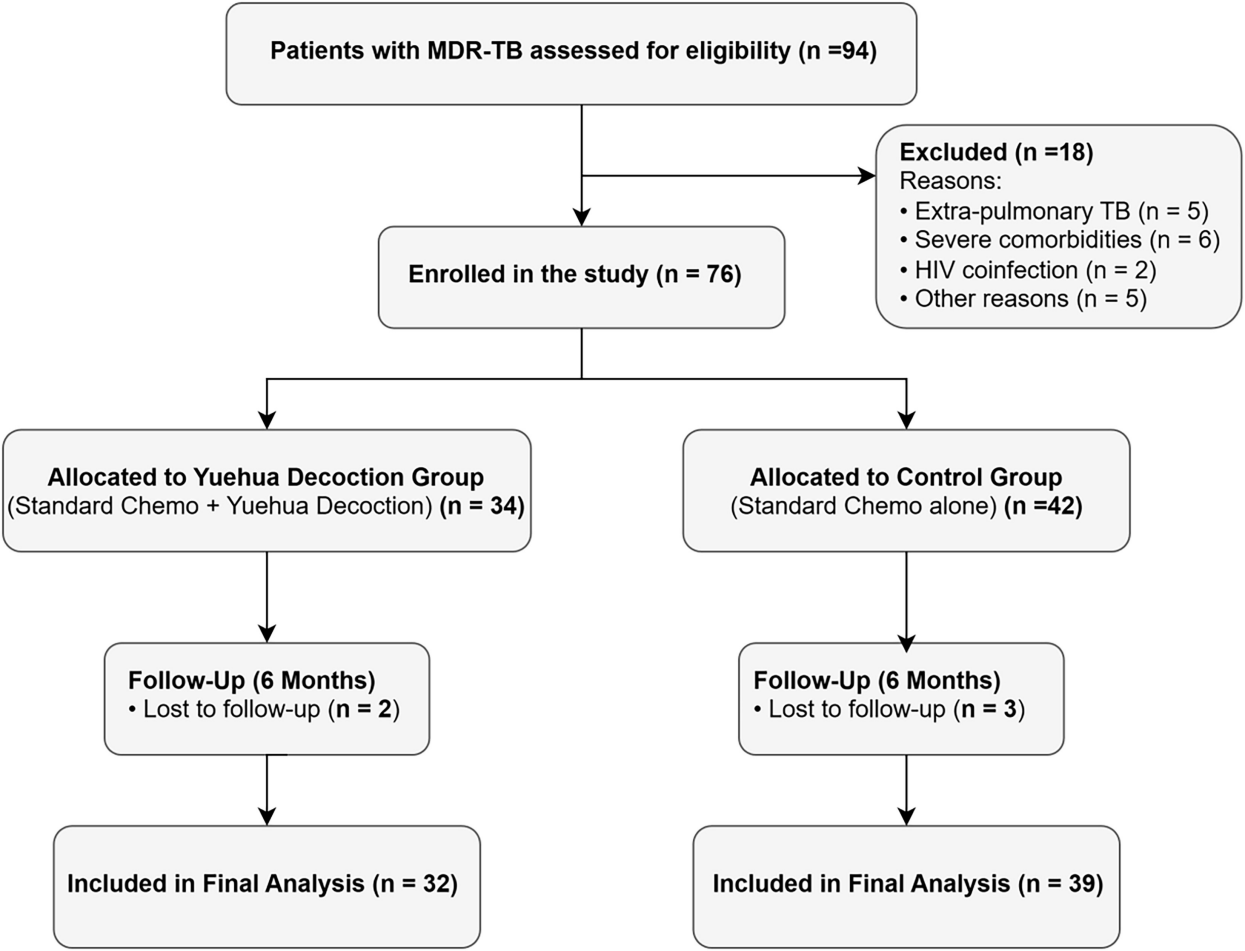
Study flow diagram. MDR-TB, multidrug-resistant tuberculosis.

A total of 94 patients with MDR-TB were screened, of whom 76 met eligibility criteria (Figure 1). Five individuals lacked complete follow-up data (2 in the Yuehua group, 3 in the control group), resulting in 71 evaluable participants (Yuehua Decoction, *n* = 32; control, *n* = 39).

Baseline demographic and clinical characteristics are presented in Table 1, including standardized mean differences (SMDs) for all variables. No significant between-group differences were observed in age, sex, BMI, ethnicity, pulmonary cavity prevalence, radiographic lesion extent, sputum smear grading, Xpert Ct values, or background chemotherapy regimens (all *P* > 0.05). However, several variables showed SMDs between 0.20 and 0.35 despite statistical non-significance (age, BMI, pulmonary cavity, lesion extent, Xpert Ct value, and fluoroquinolone use), indicating modest numerical imbalance. Age was included in all adjusted models; residual confounding by the remaining variables cannot be fully excluded and is noted in the Limitations. Two variables demonstrated meaningful imbalance: baseline SGRQ total score was higher in the Yuehua group (40.3 ± 3.2 vs. 38.1 ± 3.5; *P* = 0.007; SMD = 0.66), and diabetes mellitus was more common in the control group (35.9% vs. 15.6%; *P* = 0.065; SMD = 0.47). Both covariates were included in all adjusted models as specified in the Statistical Analysis section.

**TABLE 1:** Baseline demographic and clinical characteristics of the study population.

Characteristics	Yuehua Decoction group (*N* = 32)	Control group (*N* = 39)	*P*-value	SMD^a^
Demographics				
Age, years (Mean ± SD)	52.9 ± 16.3	47.8 ± 16.7	0.205	0.31
Male sex, n (%)	29 (90.6)	34 (87.2)	0.722	0.11
BMI, kg/m^2^ (Mean ± SD)	19.0 ± 3.1	19.7 ± 3.2	0.356	0.22
Ethnicity, n (%)	–	–	0.467	–^b^
Han	15 (46.9)	21 (53.8)		
Zhuang	13 (40.6)	10 (25.6)		
Other (Yao, Bai)	4 (12.5)	8 (20.5)		
Clinical characteristics				
Diabetes mellitus, n (%)	5 (15.6)	14 (35.9)	0.065	0.47
Pulmonary cavity, n (%)	23 (71.9)	33 (84.6)	0.247	0.31
Lesion lobes count (Mean ± SD)	3.4 ± 1.2	3.8 ± 1.5	0.143	0.29
Baseline TB severity				
Sputum smear grade, n (%)			0.277	0.12^c^
Negative/Scanty	10 (31.2)	10 (25.6)		
Positive (1+ to 4+)	22 (68.8)	29 (74.4)		
Xpert MTB/RIF Ct value (Mean ± SD)	2.4 ± 1.2	2.8 ± 1.3	0.099	0.32
Baseline quality of life				
SGRQ Total Score (Mean ± SD)	40.3 ± 3.2	38.1 ± 3.5	0.007*	0.66
Background regimen, n (%)				
Fluoroquinolones	30 (93.8)	32 (82.1)	0.171	0.35
Bedaquiline	9 (28.1)	10 (25.6)	>0.999	0.06
Linezolid	31 (96.9)	37 (94.9)	>0.999	0.10

*P < 0.05 indicates statistically significant difference.

*^a^*SMD: standardized mean difference. Continuous variables: Cohen’s *d* (pooled SD method); binary variables: difference in proportions divided by pooled SD. Values ≥ 0.20 indicate meaningful imbalance.

*^b^*SMD for categorical variables with ≥ 3 levels not directly comparable; overall chi-square *P*-value reported instead.

*^c^*SMD calculated for the positive sputum smear category vs. negative/scanty.

### Primary outcome: health-related quality of life

Table 2 summarizes the primary outcome analysis. At 6 months, SGRQ total scores were 30.4 ± 3.8 in the Yuehua group and 32.0 ± 4.1 in the control group, corresponding to mean reductions from baseline of 10.0 ± 3.1 and 6.1 ± 2.9 points, respectively. In unadjusted comparisons, the between-group difference at 6 months did not reach statistical significance (*P* = 0.299), likely reflecting the baseline imbalance in which the Yuehua group started with significantly worse SGRQ scores.

**TABLE 2:** Unadjusted and multivariable-adjusted analyses of clinical outcomes.

Outcome	Unadjusted analysis^a^ effect estimate (95% CI)	*P*-value	Adjusted analysis^b^ effect estimate (95% CI)	*P*-value
Primary outcome (linear regression)				
SGRQ score at 6 months				
Yuehua group (vs. control)	–0.94 (–2.74 to 0.85)	0.299	–3.00 (–3.87 to –2.12)	< 0.001
Secondary outcomes (logistic regression)				
Sputum culture conversion				
Yuehua group (vs. control)	1.68 (0.14–19.37)	0.679	1.08 (0.08–14.16)	0.952
Treatment success				
Yuehua group (vs. control)	1.49 (0.48–4.68)	0.491	1.36 (0.42–4.45)	0.609

^a^Unadjusted models included only the treatment group as the independent variable.

^b^Multivariable models were adjusted for age and diabetes status. The primary outcome model (SGRQ) was additionally adjusted for the baseline SGRQ score. SGRQ, St. George’s Respiratory Questionnaire; CI, confidence interval.

**FIGURE 2:**
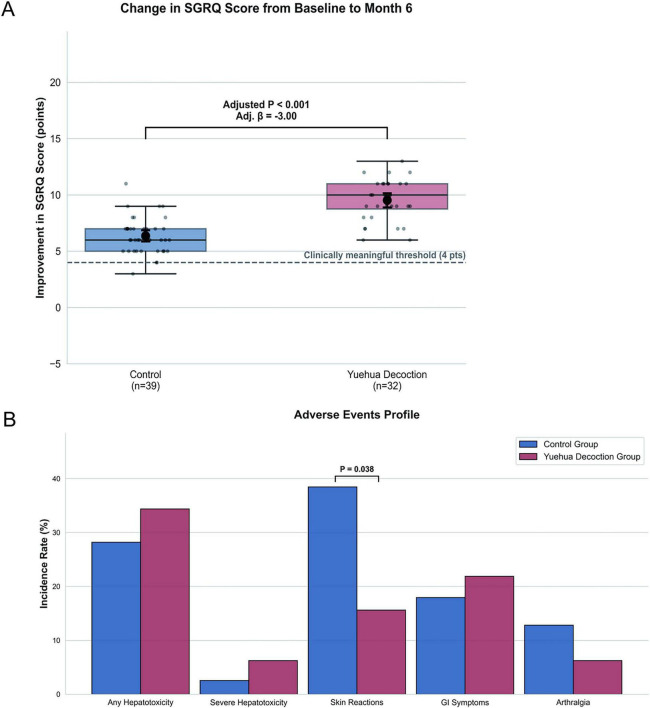
Clinical efficacy and safety outcomes. **(A)** Improvement in SGRQ total scores from baseline to Month 6. Patients receiving Yuehua Decoction showed greater quality-of-life improvement than controls (adjusted *P* < 0.001). The dashed line marks the 4-point clinically meaningful threshold. **(B)** Incidence of common adverse events during treatment. Overall safety was comparable between groups, with fewer skin reactions observed in the Yuehua Decoction group (*P* = 0.038). Sample sizes: Yuehua Decoction (*n* = 32), Control (*n* = 39). SGRQ, St. George’s Respiratory Questionnaire.

After adjustment for age, diabetes status, and baseline SGRQ score, adjunctive Yuehua Decoction was associated with a greater reduction in SGRQ total score at Month 6 (adjusted β = –3.00; 95% CI: –3.87 to –2.12; *P* < 0.001; Figure 2A). The adjusted between-group difference of 3 points falls slightly below the conventional 4-point MCID established for the SGRQ in COPD populations (29); however, this threshold has not been formally validated in MDR-TB, and the precision of the estimate (the entire 95% CI excluding zero) supports potential clinical relevance. These findings should be considered hypothesis-generating and require confirmation in a larger, blinded trial.

Results of sensitivity analyses were consistent with the primary finding. Multiple imputation yielded a pooled adjusted β of –2.89 (95% CI: –3.82 to –1.96; P < 0.001), and the worst-case analysis preserved the direction of the association (Supplementary Table 3).

### Secondary outcomes: microbiological and clinical response

Both groups achieved high rates of sputum culture conversion and treatment success (Table 2). Culture conversion was attained in 31 of 32 (96.9%) Yuehua group patients and 37 of 39 (94.9%) control group patients. Treatment success rates were 81.2% (26/32) and 74.4% (29/39), respectively. In multivariable logistic regression, point estimates favored the Yuehua group for both culture conversion (adjusted OR = 1.08; 95% CI: 0.08–14.16; *P* = 0.952) and treatment success (adjusted OR = 1.36; 95% CI: 0.42–4.45; *P* = 0.609). However, the very wide confidence intervals indicate that the study was underpowered to detect between-group differences in these outcomes, given the limited number of unfavorable events (ceiling effect).

### Safety and tolerability

The incidence and distribution of adverse events are summarized in Table 3, including event counts with denominators, CTCAE grade breakdown (Grade 1–2 vs. Grade ≥ 3), and absolute risk differences (ARDs) with 95% CIs. Both groups followed identical visit schedules and active adverse event ascertainment protocols. The overall burden of adverse events was comparable between groups, and no new safety signals were identified.

**TABLE 3:** Incidence of adverse events, CTCAE grade breakdown, and absolute risk differences.

Event	Yuehua Group (*N* = 32) n/N (%)	Control group (*N* = 39) n/N (%)	ARD (95% CI)	*P*-value^d^
Adverse events, n (%)				
Any hepatotoxicity	11/32 (34.4)	11/39 (28.2)	+6.2% (–15.5% to +27.9%)	0.614
Grade 1–2 (any grade minus ≥ 3)	9/32 (28.1)	10/39 (25.6)	+2.5% (–17.4% to +22.4%)	–
Grade ≥ 3 (severe)	2/32 (6.2)	1/39 (2.6)	+3.7% (–6.1% to +13.4%)	0.585
Skin reactions	5/32 (15.6)	14/39 (35.9)	–20.3% (–39.9% to –0.7%)	0.038
Gastrointestinal symptoms	7/32 (21.9)	7/39 (17.9)	+3.9% (–14.8% to 22.6%)	0.768
Arthralgia	2/32 (6.3)	5/39 (12.8)	–6.6% (–20.0% to 6.9%)	0.446
Clinical consequences, n (%)				
Dose reduction due to AE	8/32 (25.0)	4/39 (10.3)	+14.7% (–3.0% to 32.5%)	0.121
Treatment discontinuation due to AE	2/32 (6.3)	1/39 (2.6)	+3.7% (–6.1% to 13.4%)	0.585

ARD, absolute risk difference (Yuehua minus Control); 95% CIs calculated using normal approximation method.

^d^*P*-values calculated using Fisher’s exact test. AE, adverse event; ARD, absolute risk difference; CI, confidence interval; CTCAE, Common Terminology, Criteria for Adverse Events.

Regarding hepatotoxicity, any-grade liver injury occurred in 11 of 32 (34.4%) Yuehua group patients and 11 of 39 (28.2%) control group patients (ARD = 6.2%; 95% CI: -14.8% to 27.1%; *P* = 0.614). Severe hepatotoxicity (Grade ≥ 3) was uncommon in both groups (6.2% vs. 2.6%; *P* = 0.585). The Yuehua group had a significantly lower incidence of skin reactions [5/32 (15.6%) vs. 14/39 (35.9%); ARD = -20.3%; 95% CI: -39.3% to -1.3%; *P* = 0.038; Figure 2B]. Gastrointestinal symptoms (21.9% vs. 18.0%; *P* = 0.768) and arthralgia (6.2% vs. 12.8%; *P* = 0.439) did not differ significantly between groups. No participant in either group discontinued treatment due to hepatic adverse events.

Rates of adverse event–related dose reduction (25.0% vs. 10.3%; *P* = 0.121) and regimen discontinuation (6.2% vs. 2.6%; *P* = 0.585) did not differ significantly between groups.

## Discussion

In this prospective cohort study, adjunctive Yuehua Decoction was associated with a greater reduction in SGRQ total scores at 6 months among patients receiving contemporary MDR-TB therapy, after adjustment for baseline SGRQ score, age, and diabetes mellitus. The adjusted between-group difference of 3 points fell slightly below the conventional 4-point MCID established for the SGRQ in COPD populations (29) and the applicability of this threshold to MDR-TB has not been formally validated. Nonetheless, the precision of the estimate (95% CI: -3.87 to -2.12) and its consistency across sensitivity analyses suggest that the observed association may have clinical relevance, though confirmation in a larger, blinded trial is required.

Our findings add to the growing evidence exploring the role of TCM within the host-directed therapy (HDT) framework in tuberculosis management. Prior observational studies have suggested that Yin-nourishing herbal formulas may alleviate constitutional symptoms in pulmonary tuberculosis (16). However, most earlier investigations were limited by small sample sizes or lacked rigorous adjustment for confounding variables such as diabetes, which is a recognized risk factor for poor treatment response and prolonged symptom burden in MDR-TB (25). By adjusting for diabetes status and baseline SGRQ scores, our study provides more controlled evidence than previously available. Nevertheless, the observed association between Yuehua Decoction and improved HRQoL should be interpreted with caution, as residual confounding by unmeasured variables—including health-seeking behavior, treatment motivation, and socioeconomic factors—cannot be excluded from this non-randomized design.

Both groups achieved high rates of sputum culture conversion (>93%) and treatment success, consistent with the potent bactericidal activity of bedaquiline-based regimens reported in TB-PRACTECAL and ZeNix (32, 33). However, direct comparison with these pivotal trials is limited by differences in study design (observational vs. randomized), population characteristics (single-center Chinese cohort vs. multinational), endpoint definitions, and follow-up duration. The uniformly high success rates in our cohort may also reflect selection effects, including single-center recruitment with close adherence support and exclusion of patients with severe comorbidities or HIV coinfection; these rates may not generalize to programmatic settings. This ceiling effect limited the statistical power to detect any additional microbiological benefit from adjunctive therapy. The findings are consistent with the hypothesis that Yuehua Decoction may exert host-modulatory rather than direct antimicrobial effects, though this cannot be established from an observational study.

The safety profile of Yuehua Decoction was generally comparable to standard therapy. Concerns regarding potential hepatotoxicity often limit the use of herbal adjuncts in MDR-TB; however, severe liver injury (Grade ≥ 3) remained rare in both groups (6.2% vs. 2.6%) and comparable to global reports for bedaquiline- and linezolid-based regimens (34). No participant discontinued therapy due to hepatic adverse events.

The lower incidence of skin reactions in the Yuehua group (15.6% vs. 35.9%; ARD = –20.3%) is noteworthy, as dermatologic toxicity is a recognized barrier to treatment adherence in MDR-TB (25). This finding is biologically plausible: Ophiopogon japonicus and Stemona sessilifolia, key constituents of the formula, have been shown to modulate histamine release and hypersensitivity-related inflammatory pathways in preclinical models (23, 24). However, the absence of blinding means that differential reporting of subjective skin complaints cannot be ruled out, and this association requires confirmation in a controlled trial. Rates of adverse event–related dose reduction and regimen discontinuation did not differ significantly between groups, suggesting that the addition of Yuehua Decoction did not compromise the tolerability of the standard regimen.

Modern pharmacological studies support the biological plausibility of the observed associations. Key constituents of the formula, including Glehnia littoralis and Ophiopogon japonicus, have been shown to suppress pro-inflammatory cytokines such as TNF-α and IL-6, inhibit NF-κB signaling, and mitigate oxidative stress through Nrf2 pathway activation (35–37). These mechanisms align with current HDT strategies aimed at reducing lung tissue damage, controlling systemic inflammation, and enhancing immunologic resilience without increasing bacterial burden (12). Whether early immune modulation during the intensive treatment phase contributes to the sustained HRQoL benefits observed at 6 months remains speculative and warrants investigation through longitudinal biomarker studies.

This study has several strengths, including prospective data collection, use of a validated patient-reported outcome measure, active adverse event monitoring with standardized grading (CTCAE v5.0), and analytical control for clinically relevant confounders in a real-world population.

Several limitations should be considered when interpreting these findings. First, the non-randomized design means that confounding by indication is a central threat to internal validity; despite multivariable adjustment, residual confounding by unmeasured factors cannot be excluded. Second, the complete-case analysis excluded five patients with missing follow-up data; while sensitivity analyses using multiple imputation and worst-case assumptions yielded consistent results, bias from informative dropout cannot be fully ruled out. Third, the modest sample size (*n* = 71) limited the number of covariates that could be included in regression models. Although the primary model’s events-per-variable ratio exceeded recommended thresholds, several variables with SMDs of 0.22–0.35 (BMI, pulmonary cavity, lesion extent, Xpert Ct value, and fluoroquinolone use) were not adjusted for, representing potential sources of residual confounding. The secondary logistic regression models were more severely affected, with an EPV of 1.0 for sputum culture conversion owing to uniformly high success rates; these analyses are therefore considered exploratory and their estimates should be interpreted with caution. Fourth, the SGRQ was administered without blinding: both patients and assessors were aware of treatment assignment, introducing potential expectation bias and reporting bias for this subjective endpoint. Fifth, while the Yuehua Decoction composition was standardized by hospital SOP, formal batch-to-batch chemical fingerprinting was not performed, limiting reproducibility across sites. Sixth, this single-center study from Guangxi, China, may not generalize to other geographic settings, populations, or MDR-TB treatment regimens. These considerations underscore that the present findings are hypothesis-generating and should not be taken as evidence of a causal effect.

Although the current evidence does not support definitive clinical recommendations, our findings provide a rationale for further investigation of Yuehua Decoction as a host-directed adjunct in MDR-TB care. A multicenter, double-blind, placebo-controlled randomized trial would be the logical next step. Key design features should include: a matched placebo decoction to enable blinding; blinded outcome assessors for HRQoL evaluation; SGRQ total score change from baseline as the primary endpoint, with sample size powered to detect the 4-point MCID; HPLC-based chemical fingerprinting to ensure batch-to-batch consistency and enable cross-site replication; a pharmacokinetic sub-study to evaluate potential interactions between Yuehua Decoction constituents and bedaquiline/linezolid; and extended follow-up beyond 6 months to assess durability of any HRQoL benefit and long-term safety.

## Conclusion

In this preliminary prospective cohort study, adjunctive Yuehua Decoction was associated with improved SGRQ scores at 6 months among patients receiving standardized MDR-TB chemotherapy, after adjustment for baseline imbalances. Microbiological outcomes were comparable between groups and consistent with global benchmarks for bedaquiline-based regimens. The addition of Yuehua Decoction was associated with a lower incidence of dermatological adverse events and did not compromise regimen tolerability. These findings are hypothesis-generating and are consistent with a possible role for Yuehua Decoction as a host-directed adjunct in MDR-TB care. Confirmation in adequately powered, multicenter randomized controlled trials with blinded HRQoL outcome assessment is needed before any clinical recommendations can be made.

## Data Availability

The raw data supporting the conclusions of this article will be made available by the authors, without undue reservation.
