# Supplementary material for: Impact of Yuehua Decoction on quality of life and safety in multidrug-resistant tuberculosis: a prospective cohort study using multivariable regression analysis
Source: Front Med (Lausanne). 2026 Apr 16;13:1801335. doi: 10.3389/fmed.2026.1801335 (PMC13128372; doi:10.3389/fmed.2026.1801335)
Supplement: Supplementary file 1 [file Supplementary_file_1.DOCX]

**Supplementary Table S1. Full Model Output for SGRQ Total Score at 6 Months**

| **Variable** | **Coefficient (β)** | **Standard Error (SE)** | **P-value** | **Variance Inflation** **Factor (VIF)** |
| --- | --- | --- | --- | --- |
| Intercept | −6.22 | 2.30 | 0.009 | — |
| Yuehua Group | −3.00 | 0.44 | < 0.001 | 1.18 |
| Baseline SGRQ Score | 1.02 | 0.06 | < 0.001 | 1.19 |
| Age | −0.02 | 0.01 | 0.093 | 1.09 |
| Diabetes Mellitus | 0.53 | 0.47 | 0.255 | 1.06 |

**Supplementary Table S2. Missingness by Group and Variable**

| **Variable** | **Yuehua Group** **(N = 34 Enrolled)** | **Control Group** **(N = 42 Enrolled)** | **Total Missing** **(N = 76)** |
| --- | --- | --- | --- |
| SGRQ Total Score at 6 Months | 2 (5.9%) | 3 (7.1%) | 5 (6.6%) |
| Sputum Culture Conversion at 6 Months | 2 (5.9%) | 3 (7.1%) | 5 (6.6%) |
| Treatment Outcome at 6 Months | 2 (5.9%) | 3 (7.1%) | 5 (6.6%) |

**Supplementary Table S3. Sensitivity Analyses for Primary Outcome (SGRQ Total Score at 6 Months)**

| **Analysis Model** | **Adjusted** **Difference (β)** | **95% Confidence** **Interval** | **P-value** |
| --- | --- | --- | --- |
| Primary Analysis (Complete-Case, N = 71) | −3.00 | −3.87 to −2.12 | < 0.001 |
| Multiple Imputation by Chained Equations (MICE, M = 20) | −2.89 | −3.82 to −1.96 | < 0.001 |
| Worst-Case Assumption Analysis | −1.13 | −2.11 to −0.15 | 0.024 |
